# Mitochondrial Quality Control in Neurodegenerative Diseases: Focus on Parkinson's Disease and Huntington's Disease

**DOI:** 10.3389/fnins.2018.00342

**Published:** 2018-05-23

**Authors:** Sandra Franco-Iborra, Miquel Vila, Celine Perier

**Affiliations:** ^1^Vall d'Hebron Research Institute (VHIR)-Center for Networked Biomedical Research in Neurodegenerative Diseases (CIBERNED), Barcelona, Spain; ^2^Catalan Institution for Research and Advanced Studies, Barcelona, Spain; ^3^Department of Biochemistry and Molecular Biology, Autonomous University of Barcelona, Barcelona, Spain

**Keywords:** Parkinson's disease, Huntington's disease, mitochondrial quality control, mitochondrial dysfunction, neurodegeneration

## Abstract

In recent years, several important advances have been made in our understanding of the pathways that lead to cell dysfunction and death in Parkinson's disease (PD) and Huntington's disease (HD). Despite distinct clinical and pathological features, these two neurodegenerative diseases share critical processes, such as the presence of misfolded and/or aggregated proteins, oxidative stress, and mitochondrial anomalies. Even though the mitochondria are commonly regarded as the “powerhouses” of the cell, they are involved in a multitude of cellular events such as heme metabolism, calcium homeostasis, and apoptosis. Disruption of mitochondrial homeostasis and subsequent mitochondrial dysfunction play a key role in the pathophysiology of neurodegenerative diseases, further highlighting the importance of these organelles, especially in neurons. The maintenance of mitochondrial integrity through different surveillance mechanisms is thus critical for neuron survival. Mitochondria display a wide range of quality control mechanisms, from the molecular to the organellar level. Interestingly, many of these lines of defense have been found to be altered in neurodegenerative diseases such as PD and HD. Current knowledge and further elucidation of the novel pathways that protect the cell through mitochondrial quality control may offer unique opportunities for disease therapy in situations where ongoing mitochondrial damage occurs. In this review, we discuss the involvement of mitochondrial dysfunction in neurodegeneration with a special focus on the recent findings regarding mitochondrial quality control pathways, beyond the classical effects of increased production of reactive oxygen species (ROS) and bioenergetic alterations. We also discuss how disturbances in these processes underlie the pathophysiology of neurodegenerative disorders such as PD and HD.

## Mitochondrial dysfunction: a common feature in neurodegenerative disorders

Despite distinct clinical and pathological features, neurodegenerative diseases share critical processes such as (i) the presence of misfolded and/or aggregated proteins; (ii) neuroinflammation; (iii) impairment of autophagy; (iv) oxidative stress; and (v) mitochondrial anomalies. Neurons are highly dependent on mitochondrial function to maintain energy-intensive functions such as membrane excitability, neurotransmission, and plasticity: the brain consumes 20% of the total oxygen and about a quarter of the total glucose used by the body for energy supply.

### Parkinson's disease

Parkinson's disease (PD) is the second most prevalent neurodegenerative disorder, affecting 1–3% of the population over 65 years of age. Most PD patients are diagnosed as sporadic patients with idiopathic PD, occurring from complex interactions between environmental and genetic factors. The main features of this pathology are motor alterations such as resting tremor, postural instability, rigidity and bradykinesia, and non-motor symptoms like fatigue, depression, anxiety, sleep and autonomic disturbances, decreased motivation, apathy, decline in cognition and dementia. The neuropathological hallmarks of PD include loss of dopaminergic neurons in the midbrain substantia nigra pars compacta (SNpc) and accumulation of Lewy bodies (LBs) containing α-synuclein.

**Figure 1:**
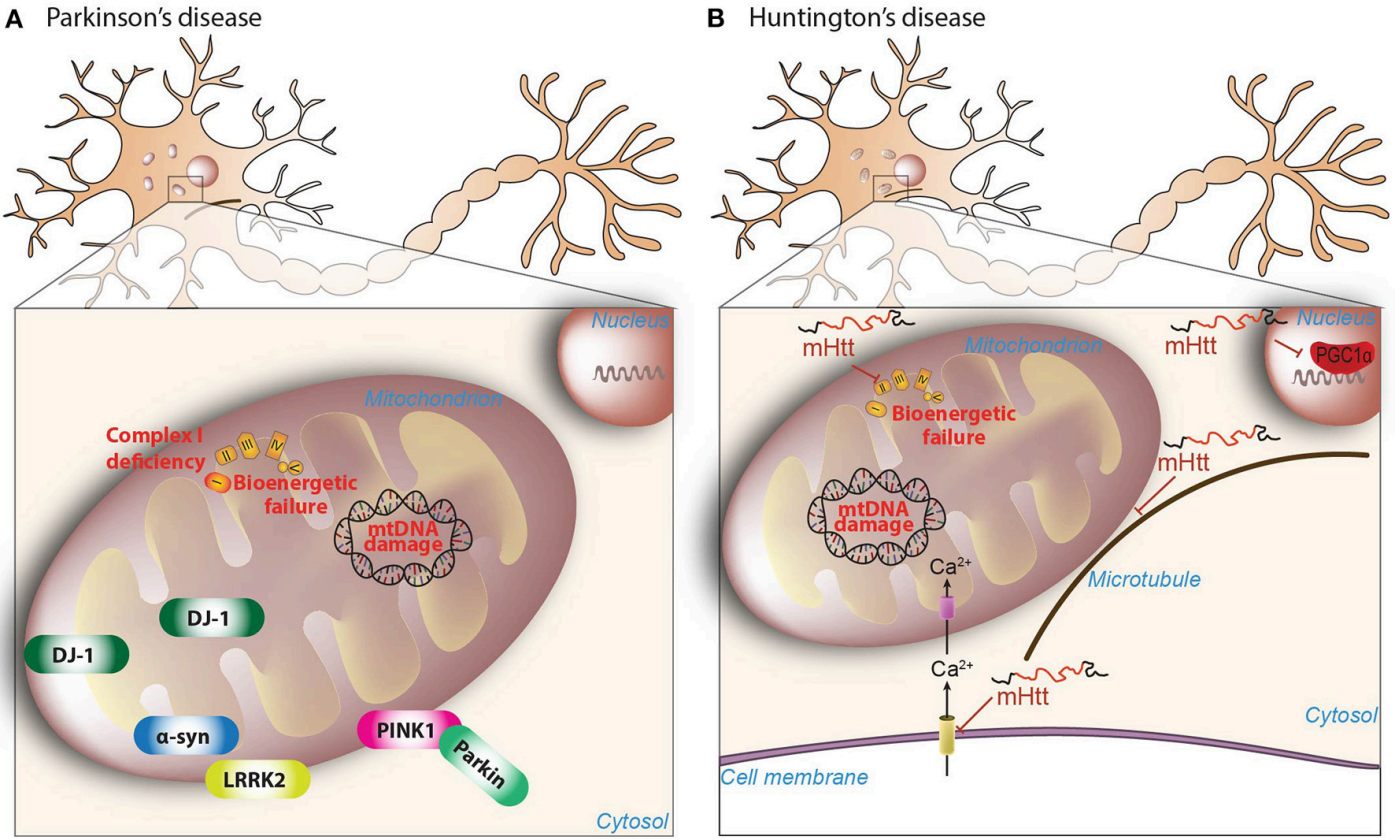
Mitochondrial dysfunction in PD and HD. **(A)** Reduced complex I activity is a classical hallmark of the pathogenesis of PD, with a subsequent increased production of mitochondrial-derived ROS. Increased ROS is accompanied by mtDNA damage and bioenergetic failure. Further highlighting the role of mitochondrial dysfunction in PD, many of the mutated nuclear genes linked to familial forms of the disease, including PINK1 (phosphatase, and tensin homolog-induced kinase 1), Parkin, α-synuclein, DJ-1, and LRRK2 (leucine-rich-repeat kinase 2), have been shown to affect many of these mitochondrial features (see main text for details). **(B)** Mutant huntingtin (mHtt) inhibits the activity of succinate dehydrogenase enzyme of complex-II of mitochondria, which may account for the bioenergetic deficit present in HD. Moreover, mHtt protein directly impairs the ability of PGC-1α to activate downstream target genes involved in mitochondrial biogenesis and normal mitochondrial function. Further amplifying the damage, mHtt is able to promote calcium-dependent mitochondrial depolarization and mitochondrial swelling. Mitochondrial transport is also impaired due to the ablity of mHtt to combines specifically with the beta subunit of tubulin.

The first evidence linking mitochondrial dysfunction and PD was the discovery that MPTP (1-methyl-4-phenyl-1,2,3,6-tetrahydropyridine), an inhibitor of complex I (NADH/ubiquinone oxidoreductase) of the mitochondrial electron transport chain, causes parkinsonism in humans (Langston et al., 1983). Later, sporadic PD patients were reported to present reduced complex I activity, not only in the SNpc (Schapira et al., 1990) but also in other brain areas and peripheral tissues (Parker et al., 1989, 2008; Krige et al., 1992; Blin et al., 1994; Haas et al., 1995), as well as in cytoplasmic hybrid (cybrid) cells (Swerdlow et al., 1996) derived from PD patients (Figure 1). Besides the mitochondrial complex I defect, genetic evidence suggests that mutations in mitochondrial DNA (mtDNA) also play a role in the pathogenesis of PD. Haplogroups J and K have a protective effect against PD in certain European populations (van der Walt et al., 2003; Fachal et al., 2015), whereas haplogroup HV has been reported to increase the risk of PD in Caucasians (Hudson et al., 2013). Alterations in mtDNA have long been hypothesized to play a pathogenic role in PD. In particular, multiple mtDNA deletions have been observed in SNpc dopaminergic neurons from post-mortem human brains of both aged individuals and patients with idiopathic PD (Bender et al., 2006; Kraytsberg et al., 2006). Mutations in mitochondrial DNA polymerase gamma (*POLG*), which result in the accumulation of multiple mtDNA deletions in muscle, have been associated with levodopa-responsive parkinsonism with severe SNpc dopaminergic neuronal loss, usually as part of a more complex syndrome (Luoma et al., 2004; Davidzon et al., 2006). Our group and others showed that despite high levels of mtDNA deletions, SNpc dopaminergic neurons from *POLG* mutant mice did not exhibit gross mitochondrial dysfunction or degeneration (Perier et al., 2013; Dai et al., 2014), indicating that a high level of mtDNA deletions is not sufficient *per se* to trigger cell death in SNpc dopaminergic neurons. In contrast, partial depletion of mtDNA in mice by a conditional disruption in dopaminergic neurons of mitochondrial transcription factor A (TFAM), which regulates mtDNA transcription, leads to a decrease in mtDNA content and cytochrome c oxidase enzymatic activity, which is associated with a progressive parkinsonism phenotype (Ekstrand et al., 2007). These findings strongly support a role of respiratory chain and mitochondrial dysfunction in the pathogenesis of PD.

A primary role of mitochondrial dysfunction in this process was boosted by the identification of genes related to familial forms of PD: α-synuclein (*SNCA*), leucine-rich repeat kinase 2 (*LRRK2*), PTEN-induced putative kinase 1 (*PINK1*), parkin RBR E3 ubiquitin protein ligase, also known as Parkin, (*PRKN*), DJ-1 (*PARK7*), and vacuolar sorting protein 35 (*VPS35*; Figure 1). Mutations in *SNCA, LRRK2*, and *VPS35* are associated with autosomal dominant PD. Several missense mutations have been linked to α-synuclein (A53T, A30P, E46K, H50Q, and A53E) and some families also present duplications and triplications of the *SNCA* wild-type gene (Polymeropoulos et al., 1997; Krüger et al., 1998; Singleton et al., 2003; Zarranz et al., 2004; Appel-Cresswell et al., 2013; Proukakis et al., 2013; Pasanen et al., 2014). *SNCA* mutations have been linked to increased mtDNA damage, mitophagy, and mitochondrial fission, among others (Wong and Krainc, 2017). For example, the A53T mutation leads to increased mitochondrial fission (Xie and Chung, 2012; Pozo Devoto et al., 2017) and mitophagy (Chinta et al., 2010; Choubey et al., 2011; Chen et al., 2015). *LRRK2* mutations are the major cause of familial PD (Paisán-Ruiz et al., 2004; Zimprich et al., 2004), with more than 50 mutations identified thus far, the most common of which affects the kinase activity of the protein (Nuytemans et al., 2010). *LRRK2* mutations in *Caenorhabditis elegans* and neurons derived from induced pluripotent stem cells are associated with mitochondrial dysfunction and altered mitochondrial dynamics (Saha et al., 2009; Wang et al., 2012). Mutations in *VPS35* have been recently described (Vilariño-Güell et al., 2011; Zimprich et al., 2011), and may cause abnormal trafficking of cargo by mitochondrial-derived vesicles (MDVs) (Wang et al., 2015), these being cargo-selective vesicles that bud off mitochondria independently from the mitochondrial fission machinery (Neuspiel et al., 2008).

PINK1, a serine/threonine kinase, and Parkin, an E3 ubiquitin ligase, work together in a common pathway leading to the clearance of dysfunctional mitochondria (Pickrell and Youle, 2015). Mutations in *PINK1* and Parkin gene are associated with recessive PD (Kitada et al., 1998; Valente et al., 2004). Parkin loss in mice and Drosophila flies leads to decreased complex I and complex IV activities (Palacino et al., 2004; Casarejos et al., 2006), with Drosophila flies exhibiting locomotor defects, reduced lifespan, mitochondrial abnormalities and dopaminergic neuron degeneration (Greene et al., 2003). In cell lines, loss of PINK1 or mutations therein lead to decreased mitochondrial respiration and decreased ATP synthesis (Liu et al., 2009). DJ-1 mutations also cause autosomal recessive PD (Thomas and Beal, 2007) leading to impaired mitochondrial respiration, reduced membrane potential, increased ROS levels and altered mitochondrial morphology (Krebiehl et al., 2010).

### Huntington's disease

Huntington's disease (HD) is a devastating neurodegenerative disease affecting 10.6–13.7 individuals per 100,000 people in Western populations (Bates et al., 2015). It is an autosomal dominant neurodegenerative disorder, characterized by the abnormal expansion of the cytosine, adenine, and guanine (CAG) triplet repeats in the polyglutamine region of the huntingtin (*HTT*) gene. Clinically, HD is characterized by motor dysfunction, cognitive decline, and psychiatric disturbances caused by the preferential atrophy of GABAergic medium spiny neurons in the striatum as well as in other regions such as the cerebral cortex (McColgan and Tabrizi, 2018). How the mutant huntingtin protein elicits its toxic effects remains elusive, but several lines of evidence suggest involvement of transcriptional dysregulation and impaired mitochondrial function in the pathogenesis of HD (Labbadia and Morimoto, 2013; Figure 1).

Bioenergetics has been a classical field of study in HD research since the discovery of glucose hypometabolism in the caudate nuclei of presymptomatic HD subjects (Grafton et al., 1992). Both HD patients and presymptomatic carriers present increased lactate levels in brain areas linked with the disease (Jenkins et al., 1993), which is indicative of impaired energy metabolism. Interestingly, the presence of metabolic alterations in presymptomatic carriers argues in favor of mitochondrial dysfunction as an early appearing defect in the disease. As for PD and complex I, HD is linked to reduced complex II and complex III activity in the striatum, with complex IV also being affected to a lesser extent (Brennan et al., 1985; Gu et al., 1996). Accidental intoxication is also capable of recapitulating some of the clinical and pathologic phenotypes of HD in humans (Ludolph et al., 1991). Indeed, intoxication with mildewed sugar cane contaminated with 3-nitropropionic acid (3-NPA), an inhibitor of the succinate dehydrogenase enzyme of complex II, is able to induce HD symptoms in rodents and non-human primates (Brouillet et al., 2005). Studies have also suggested the involvement of mtDNA mutations in HD pathogenesis, as an increased frequency of mtDNA lesions and mtDNA depletion are also found in the HD striatum and skin fibroblasts (Siddiqui et al., 2012). How mitochondrial dysfunction and bioenergetic defects in HD occur is still a matter of debate. Nonetheless, mutant huntingtin is capable of directly interacting with the outer mitochondrial membrane (OMM) (Gutekunst et al., 1998; Choo et al., 2004; Orr et al., 2008), reinforcing the notion that mutant huntingtin directly impairs mitochondrial function. Further linking mitochondrial dysfunction and HD is the ability of mutant huntingtin to inhibit peroxisome proliferator-activated receptor gamma coactivator 1-α (PGC-1α) expression (Figure 1). PGC-1α is a transcriptional coactivator that regulates several metabolic processes such as mitochondrial biogenesis, thermogenesis or oxidative phosphorylation (OXPHOS) (Puigserver and Spiegelman, 2003). Mutant huntingtin interferes with the PGC-1α transcriptional pathway, impairing its ability to activate downstream genes, while PGC-1α ectopic expression provides neuroprotection in transgenic HD mice and in the 3-NPA mouse model (Cui et al., 2006; Weydt et al., 2006).

Beyond bioenergetics, mitochondria also regulate calcium homeostasis. Panov et al. (2002) demonstrated that lymphoblast mitochondria from HD patients have a lower membrane potential and depolarize at lower calcium loads compared to control mitochondria. This defect was also present in brain mitochondria from a HD transgenic mouse model (Panov et al., 2002). Cyclosporine treatment, which inhibits mitochondrial permeability transition pore (mPTP) opening, was able to delay this effect (Quintanilla et al., 2013), supporting a role for dysfunctional mitochondrial calcium handling in the pathogenesis of HD.

Considering the important role that mitochondrial function plays in neuronal homeostasis, some endogenous mechanisms have evolved to maintain mitochondrial integrity and functionality. These mechanisms can be divided into different categories, and although they respond to similar cues, the degree of damage determines their sequential activation. Upon excessive production of reactive oxygen and nitrogen species, for example, the first level of defense takes place with a coordinated detoxifying network. The second level of defense maintains the integrity of the mitochondrial proteome, while the third level controls organellar shape and number through interconnected processes such as mitochondrial fusion, fission and mitophagy. In this review article, we focus attention on the role of mitochondrial quality control processes that are responsible for maintaining mitochondrial integrity and functionality, and how these mechanisms may be functionally disturbed in PD and HD.

## Mitochondria, the source, and target of ROS: mechanisms for keeping them healthy

**Figure 2:**
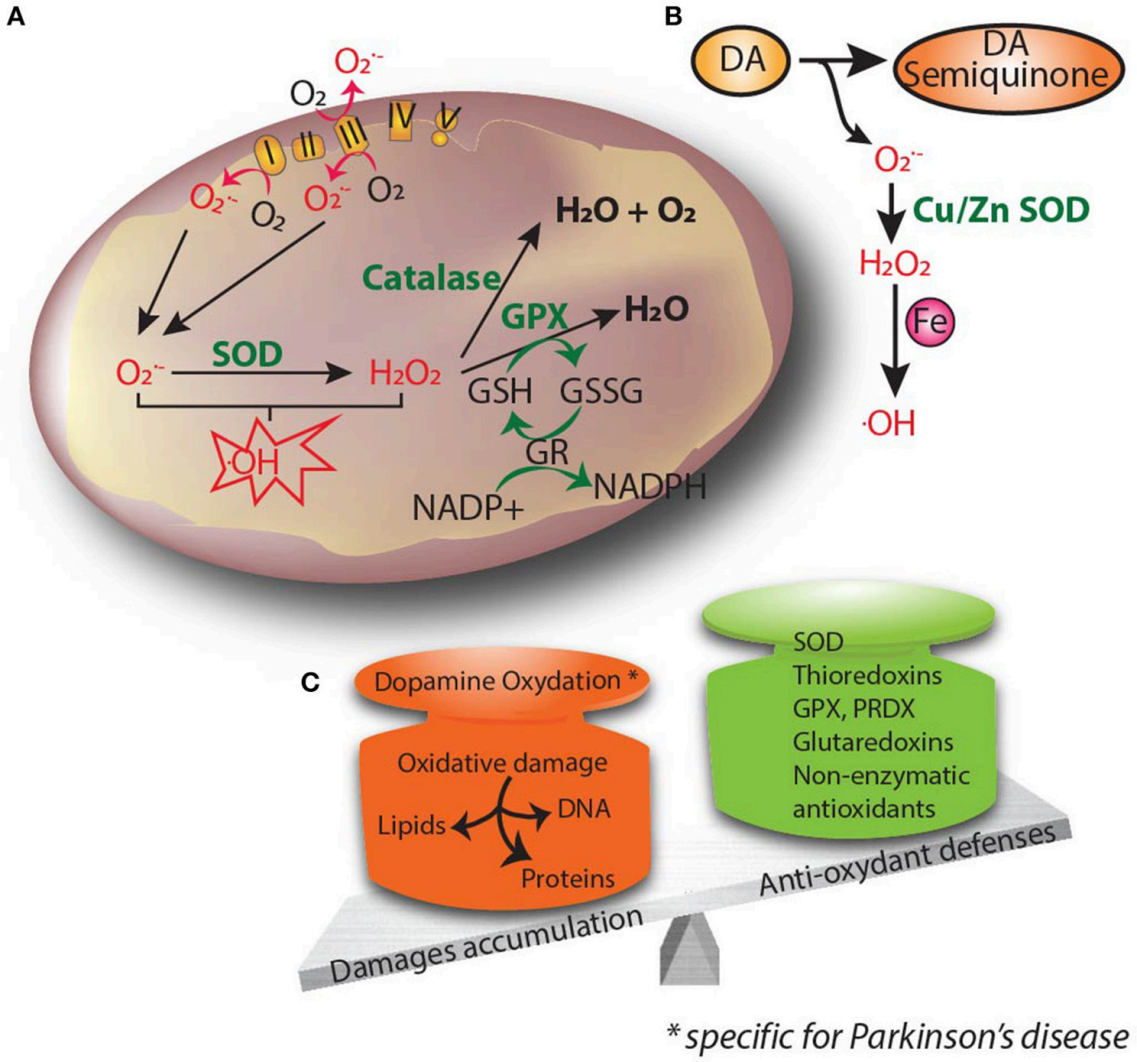
Oxidative stress is thought to be the common underlying mechanism that leads to cellular dysfunction and eventual cell death. **(A)** Oxidants and superoxide radicals are produced as by-products of oxidative phosphorylation, making mitochondria the main site of reactive oxygen species (ROS) generation within the cell. **(B)** Dopamine can be oxidized to reactive dopamine quinones, contributing to increased levels of ROS. **(C)** Oxidative stress occurs when there is an imbalance between ROS production and cellular antioxidant activity. Excessive ROS causes lipid peroxidation, DNA damage and protein oxidation. Accumulation of free radicals causes mitochondrial dysfunction and contributes to the propagation of stress related to neuronal pathophysiology.

The term oxidative stress describes conditions that result from the imbalance between free radical generation, their detoxification and elimination. Mitochondria are the main source of cellular reactive oxygen species (ROS) as well as having the highest antioxidant capacity (Figure 2). The harmful effects of ROS involve damage to macromolecules such as proteins, lipids, polysaccharides, or nucleic acids. The intrinsic properties of neurons, i.e., (i) high metabolic rates; (ii) a rich composition of fatty acids prone to peroxidation; (iii) high intracellular concentrations of transition metals, capable of catalyzing the formation of reactive hydroxyl radicals; (iv) low levels of antioxidants; and (v) reduced capability to regenerate, make them highly vulnerable to the detrimental effects of ROS. Under normal conditions, small amounts of molecular oxygen in the mitochondria are reduced to ROS rather than being converted into water. Because of its high metabolic rate, the brain consumes high levels of oxygen, leading to increased radical formation. Thus, the presence of antioxidant defenses to reduce ROS levels below a toxic threshold is fundamental. The superoxide radical (^∙^${\text{O}}_{2}^{-}$) is the first radical that appears with the reduction of molecular oxygen. It can be transformed into hydrogen peroxide (H_2_O_2_) by the action of the enzyme superoxide dismutase (SOD). Hydrogen peroxide is poorly reactive with most biomolecules, but it can react with Fe^2+^ (in the Fenton reaction) to give rise to the hydroxyl radical (OH^∙^), probably the most reactive free radical found *in vivo* (Halliwell and Gutteridge, 1984; Liochev, 2013), which can react with virtually all biomolecules. To maintain a balanced level of reactive species, cells have developed various antioxidant defenses, the first being SOD (superoxide dismutase), which accelerates the dismutation of two molecules of superoxide into one molecule of H_2_O_2_ and one molecule of O_2_, thus, reducing the potentially harmful effects of ^∙^${\text{O}}_{2}^{-}$. Three SOD isoenzymes have been identified in humans: copper and zinc-containing SOD (CuZnSOD/SOD1), mainly located in the cytosol and nuclear compartments; manganese-containing SOD (MnSOD/SOD2), a mitochondrial enzyme; and SOD3, a MnSOD located extracellularly (Perry et al., 2010). Even though H_2_O_2_ is a radical with low reactivity, it must be eliminated by the action of enzymes such as catalase, glutathione peroxidases (GPXs), the thioredoxin system, peroxiredoxins (PRDX), and glutaredoxins. GPX couples the reduction of H_2_O_2_ (and other peroxides) to H_2_O (or to corresponding alcohols) with the oxidation of reduced glutathione (GSH) to oxidized glutathione (GSSG), which can be converted back to GSH by glutathione reductase (GR). PRDX also couples the reduction of H_2_O_2_ with a cycle of peroxide-dependent oxidation and thiol-dependent reduction of cystein residues, the latter catalyzed by thioredoxin and thioredoxin reductase. Glutaredoxins have the same function as the thioredoxin system (Chance et al., 1979; Rhee et al., 2005). Glutathione S-transferases participate in the detoxification of electrophilic compounds by their conjugation with GSH (Hayes et al., 2005). Another group of non-enzymatic antioxidants such as glutathione, ascorbic acid (vitamin C), α-tocopherol (vitamin E), carotenoids, or flavonoids, which are mainly ingested with the diet, act by scavenging free radicals (Forman et al., 2014). The last antioxidant defense layer is the sequestration of “free” transition metals, such as iron or copper, which are capable of stimulating free radical formation and also the direct oxidation of macromolecules. Within cells, iron is stored in the form of ferritin and copper as metallothionein (Rutherford and Bird, 2004).

### Oxidative stress and parkinson's disease

It is now clear that complex I inhibition-induced mitochondrial dysfunction causes the chronic production of ROS and is instrumental in the demise of dopaminergic neurons. Some catalytic subunits of complex I have been found to be oxidized in PD brain (Keeney et al., 2006), which represents a feed-forward cycle in which complex I inhibition produces ROS, which in turn impacts on complex I activity. Oxidative damage to proteins, lipids and DNA has been observed in post-mortem brain samples from PD patients (Dauer and Przedborski, 2003). Supporting a pathogenic role for mitochondria-derived ROS in the context of PD, transgenic mice overexpressing human catalase (an antioxidant enzyme normally localized in the peroxisome) specifically targeted to the mitochondria exhibit an attenuation of MPTP-induced mitochondrial ROS and reduced dopaminergic cell death (Perier et al., 2010). Moreover, oxidation-mediated damage to mitochondrial components, such as optic atrophy 1 (OPA1), contributes to PD neurodegeneration by remodeling the mitochondrial structure and cristae organization. Such cristae remodeling associated with cardiolipin peroxidation induces cytochrome c release and subsequent activation of apoptosis (Perier et al., 2005; Ramonet et al., 2013).

The SNpc is particularly sensitive to oxidative stress, probably because of its unique dopaminergic environment. Dopamine is vulnerable to auto- and/or enzymatic oxidation, producing reactive dopamine quinone, and ROS (Figure 2; Graham et al., 1978; Maker et al., 1981). Dopamine-induced toxicity has been observed in several *in vitro* and *in vivo* models (Hastings et al., 1996), and has been reported to affect several proteins, among them complex I (Ben-Shachar et al., 2004). Recently, it was shown that dopaminergic neurons derived from idiopathic and familial PD patients had decreased basal respiration and accumulation of oxidized dopamine. Interestingly, the treatment of those neurons with isradipine, a Cav1 channel antagonist, or a calcineurin inhibitor was able to decrease oxidized dopamine levels (Burbulla et al., 2017), highlighting the important role of calcium in dopamine oxidation. Besides, the expression of constitutively open Cav1.3 channels has been suggested to render nigrostriatal neurons more vulnerable to excitotoxic cell death. SNpc neurons are able to generate action potentials in the absence of synaptic input (Romo and Schultz, 1990). For their pacemaking activity, SNpc neurons not only engage monovalent cation channels but also L-type calcium channels with a distinctive Cav1.3, that allow calcium entry into the cytoplasm (Ping and Shepard, 1996). The engagement of Cav1.3 calcium channels comes at a metabolic cost for the neurons, since they have to pump calcium back through ATP-dependent pumps (Wilson and Callaway, 2000). A key paper by Guzman et al. (2010) showed how L-type calcium channels induce oxidative stress specific to SNpc dopaminergic neurons, which exhibit higher levels of basal oxidation compared to dopaminergic ventral tegmental area (VTA) neurons. Antagonizing L-type calcium channels lowered the oxidation state of SNpc dopaminergic neurons, but not VTA neurons. Moreover, blocking calcium entry into the mitochondria also decreased oxidation (Guzman et al., 2010). Interestingly, oxidative stress is amplified in SNpc dopaminergic neurons from DJ-1 KO mice. These mice exhibit decreased mRNA levels of two UCPs (uncoupling protein), Ucp4 and Ucp5, in the SNpc leading to a blunted UCP-mediated mitochondrial uncoupling, which could explain the increased oxidation state (Guzman et al., 2010). Taken together, this work suggests that SNpc dopaminergic neurons have higher basal oxidation states, which is a consequence of the activation of L-type calcium channels during autonomous pacemaking. Additionally, SNpc dopaminergic neurons have also a relatively large axonal arborization (Matsuda et al., 2009), increasing their bioenergetic demands and rendering them more susceptible to ROS production. Pacelli et al. (2015) showed that SNpc neurons have a higher oxygen consumption rate and a lower maximal capacity together with increased ROS levels compared to VTA neurons, due to their larger axonal arborization. Neurons with smaller arborization size were less susceptible to die from the toxic effects of complex I inhibitors such as MPP^+^ or rotenone (Pacelli et al., 2015).

Not only are SNpc dopaminergic neurons more vulnerable to oxidative stress, but PD neuropathogenesis is associated with alterations in the antioxidant defense system (Figure 2). SOD2 activity in PD post-mortem tissue is increased, highlighting the mitochondria as one of the main sites of ROS production (Saggu et al., 1989; Poirier et al., 1994). SOD1 and α-synuclein co-deposition with Lewy pathology has also been reported (Nishiyama et al., 1995), together with an upregulation of SOD1 (Marttila et al., 1988). Degenerating neurons exhibit reduced intracellular copper levels (Davies et al., 2014), which renders the SOD1 metal-deficient isoform more prone to aggregate. This vicious circle is then fed with reduced copper metallation of SOD1, decreasing its enzymatic function on the one hand and upregulating oxidative stress-induced SOD1 on the other. As a result, SOD1 aggregates increase, rendering SNpc neurons more vulnerable to oxidative stress (Trist et al., 2017). Additionally, GSH content is decreased in the SNpc post-mortem tissue of PD patients (Sian et al., 1994). Metal homeostasis is also dysregulated in PD patients, who display a higher content of iron in the SNpc compared to age-matched controls (Dexter et al., 1989). Increased levels of iron can stimulate free radical formation through the Fenton reaction, leading to the generation of highly reactive OH^∙^ radicals. Interestingly, iron chelation was shown to be neuroprotective against MPTP-induced dopaminergic cell death (Kaur et al., 2003).

### Oxidative stress and huntington's disease

Several markers of oxidative stress were found in post-mortem tissue of HD patients; these include (i) lipofuscin accumulation, (ii) presence of the classical marker of oxidative modification in proteins (protein carbonyls) and lipids (malondialdehyde and 4-hydroxynonenal), and (iii) markers of oxidative damage to nuclear DNA, such as 8-hydroxydeoxyguanosine (Browne et al., 1997; Stack et al., 2008).

HD neuropathogenesis is also associated with alterations in the antioxidant defense system (Figure 2). Glutathione is decreased in the cerebral cortex from HD patients (Beal et al., 1992), whereas antioxidant enzymes, such as peroxiredoxin and GPX, together with the activity of SOD2 and catalase, were reported to be increased in the post-mortem human HD striatum (Sorolla et al., 2008). In general, the enhanced activity of antioxidants reduces neuronal oxidative stress by maintaining ROS at nanomolar levels, indicating that only neurons with higher antioxidant capacity are able to survive. Impairment of antioxidant defenses renders proteins, among other biomolecules, more prone to oxidation. In HD, the enzyme aconitase is one of the most affected enzymes of the tricarboxylic acid cycle (TCA), probably due to its Fe-S clusters. Aconitase oxidation is accompanied by decreased activity in the HD caudate, putamen and cerebral cortex (Tabrizi et al., 1999). Importantly, aconitase inactivation directly correlates with the generation of superoxide produced by excitotoxicity (Patel et al., 1996). Moreover, mitochondrial creatine kinase, citrate synthase, and ATP synthase are also oxidized in the striatum of HD patients, leading to a reduced catalytic activity (Sorolla et al., 2010) and providing a link between oxidative stress and the characteristic bioenergetic deficit present in HD.

Several neuroimaging studies suggest an increased iron signal in the basal ganglia of HD patients (Muller and Leavitt, 2014), which was confirmed by biochemical and histochemical evidence (Dexter et al., 1991), although the findings have some limitations. Another study identified that not only iron but also copper increases in R6/2 transgenic mice (expressing truncated N-terminal fragments of mutant huntingtin with 144 CAG repeats), which would contribute to creating a toxic microenvironment in HD pathology (Fox et al., 2007). Iron-containing proteins also become altered in HD (Muller and Leavitt, 2014). Succinate dehydrogenase, the main component of complex II, displayed decrease expression of its Fe-S subunit Ip and subunit Fp in human post-mortem tissue and in a HD cellular model. Reduced expression of those subunits was accompanied by a decrease in succinate dehydrogenase catalytic activity (Benchoua et al., 2006).

Taken together, these data highlight that antioxidant defense mechanisms are hampered in the context of PD and HD, with specific enzymes being altered in each pathology, but always leading to a scenario in which the balance between ROS production and scavenging is altered. This breakdown can have important implications in the pathology of neurodegenerative disorders, amplifying the detrimental cascade and generating new defects.

## Mitochondrial protein quality control: the guarantee of mitochondrial health

The endosymbiotic hypothesis argues that the mitochondrion evolved from a bacterial ancestor within the phylum *Alphaproteobacteria* through a symbiotic process within a eukaryotic host cell (Margulis, 1970). Mammalian mitochondria have a ~16-kb genome encoding for 13 protein subunits of the mitochondrial electron transport chain and ATP synthase, as well as the ribosomal RNA (rRNA) and transfer RNA (tRNA) components of the mitochondrial translation system (Anderson et al., 1981). This means that most of the mitochondrial proteome is encoded in the nucleus, synthesized in the cytosol, and imported into the organelle. Most nuclear-encoded mitochondrial proteins are synthesized as precursors and maintained in an unfolded conformation to make them translocation-competent. Precursor proteins in the cytosol must exist in complexes with cytosolic chaperones, such as HSP70 and HSP90, to avoid their degradation and aggregation (Young et al., 2003). Moreover, those precursors need to contain information that directs them to the mitochondria and into the correct mitochondrial compartment. The most common signal is the N-terminal targeting sequence called mitochondrial-targeting sequence (MTS), which consists of 10–80 amino acid residues with no sequence identity, but with some characteristic physicochemical properties, such as its potential to form amphipathic helices with one hydrophobic and one positively charged face (Roise et al., 1986; Pfanner et al., 2000) The MTS is normally cleaved in the matrix leading to the mature polypeptide. There are also C-terminal targeting sequences and internal signals whose purpose remains elusive (Gordon et al., 2000). Most mitochondrial proteins are located within the matrix; for these proteins to be imported, there needs to be cooperation between the two main mitochondrial translocases—the translocase of the outer membrane (TOM) complex on the outer membrane, formed by seven components (Tom70, Tom22, Tom20, Tom40, Tom5, 6, and 7) (Model et al., 2008; Bausewein et al., 2017), and the translocase of the inner membrane (TIM23) complex (Tim50, Tim17, Tim21, Tim23, Tim44, Pam 17, Tim16/Pam16, Tim14/Pam18, mtHSP70, and Mge1) (Mokranjac and Neupert, 2010). The TOM complex mediates the translocation of nearly all of the mitochondrial proteome, whereas the TIM23 complex translocates pre-proteins through the inner mitochondrial membrane. Translocation through the TIM23 complex requires an intact mitochondrial membrane potential and the hydrolysis of ATP. The inner mitochondrial membrane harbors a variety of proteins, including the mitochondrial electron transport chain complexes and ATP synthase. There are three different routes for the import of inner mitochondrial membrane proteins once they have been translocated by the TOM complex: (1) through the TIM22 complex located in the inner mitochondrial membrane, (2) through a lateral insertion by the TIM23, complex, and (3) in an export-like route from the matrix to the inner membrane (Wiedemann and Pfanner, 2017).

Changes in the stoichiometric balance between nuclear- and mitochondrial-encoded proteins due to improper mitochondrial protein import and folding, as well as mutations in mtDNA, affect organelle proteostasis (Houtkooper et al., 2013). This is sufficient to disrupt the integrity and functionality of mitochondria. Mitochondrial protein import is thus under the surveillance of molecular chaperones (proteins that stabilize or assist the acquisition of the active conformation of other proteins without being part of their final structure) in order to avoid misfolding or aggregation of the newly transported proteins (Kim et al., 2013). mtHSP70 (mitochondrial heat shock 70 kDa protein), also called mortalin, is a chaperone crucial for the trafficking of proteins into the mitochondrial matrix, since it forms the core of the import motor between the TIM23 complex and the matrix. Mortalin also interacts with HSP60 (heat shock 60 kDa protein), one of the most important components of the protein folding machinery in the matrix, helping with the proper folding of newly imported proteins (Deocaris et al., 2006). The HSP60 and HSP10 (heat shock 10 kDa protein) machineries form two stacked rings in the mitochondrial matrix that allow accommodation of the unfolded polypeptide following the hydrolysis of ATP, assisting henceforth its proper folding (Walter, 2002; Okamoto et al., 2017). TRAP1 (tumor necrosis factor receptor-associated protein 1), another molecular chaperone present in the matrix, was first identified as a member of the HSP90 (heat shock 90 kDa protein) family because of its high degree of sequence and structural similarity. HSP90 and TRAP1 both have an N-terminal targeting sequence and an ATP-binding domain, but TRAP1 displays different functional characteristics (Song et al., 1995) and is suggested to play an important role in preventing ROS-dependent cell death (Gesualdi et al., 2007). In the intermembrane space, the small TIM chaperones assist the transfer of polypeptides through this compartment (Webb et al., 2006).

Another critical component of mitochondrial quality control relies on proteolytic systems located in different mitochondrial compartments. Together with chaperones, these systems participate in the import and trafficking of proteins from the cytosol into the mitochondria. Mitochondrial processing peptidase (MPP) is the main peptidase responsible for cleaving the targeting signals of preproteins. After MPP processing, other peptidases can act, such Oct 1 (octapeptidyl aminopeptidase 1) which removes an octapeptide (Gakh et al., 2002) and Icp55 (intermediate cleaving peptidase of 55 kDa) which removes a unique amino acid to stabilize the mitochondrial proteins (Vögtle et al., 2009). Besides their role in protein trafficking through the mitochondria, mitochondrial proteases are required to degrade misfolded, damaged, and unfolded proteins that are no longer capable of being refolded by chaperones, as well as process proteins that have been imported into the mitochondria. ATP-dependent proteases hydrolyse ATP to disassemble, unfold and sequester their substrates in compartments where they are degraded. Both iAAA and mAAA (ATPases associated with diverse cellular activities) proteases are inserted into the inner mitochondrial membrane, with iAAA facing the intermembrane space and mAAA facing the matrix; these participate in OXPHOS chain subunits' quality control and turnover (Gerdes et al., 2012). LONP1 (Lon protease) and CLPP (Clp protease proteolytic subunit) are both serine peptidases acting in the mitochondrial matrix (Haynes et al., 2007; Quirós et al., 2014). LONP1 homozygous deletion in mouse causes embryonic lethality, underscoring its crucial role in cellular homeostasis (Quirós et al., 2014). Once the ATP-dependent proteases act, oligopeptidases further process the substrates. The ATP-independent proteases HTRA2 (high temperature requirement protein A2) and ATP23 are located in the intermembrane space and participate in the protein quality control of this compartment (Clausen et al., 2011; Quirós et al., 2015). These proteases are highly regulated and are able to modulate many biochemical activities such as protein importation, cardiolipin metabolism, mitochondrial gene expression, and mtDNA stability (Quirós et al., 2015). For instance, HTRA2 also has a role during apoptosis, since it can be released from the intermembrane space to relieve the inhibition of cytosolic caspases by binding to inhibitors of apoptotic proteins in the cytosol (Hegde et al., 2002; Verhagen et al., 2002). HTRA2 can also mediate caspase-independent cell death through its own protease activity (Hegde et al., 2002).

### Mitochondrial protein quality control and parkinson's disease

**Figure 3:**
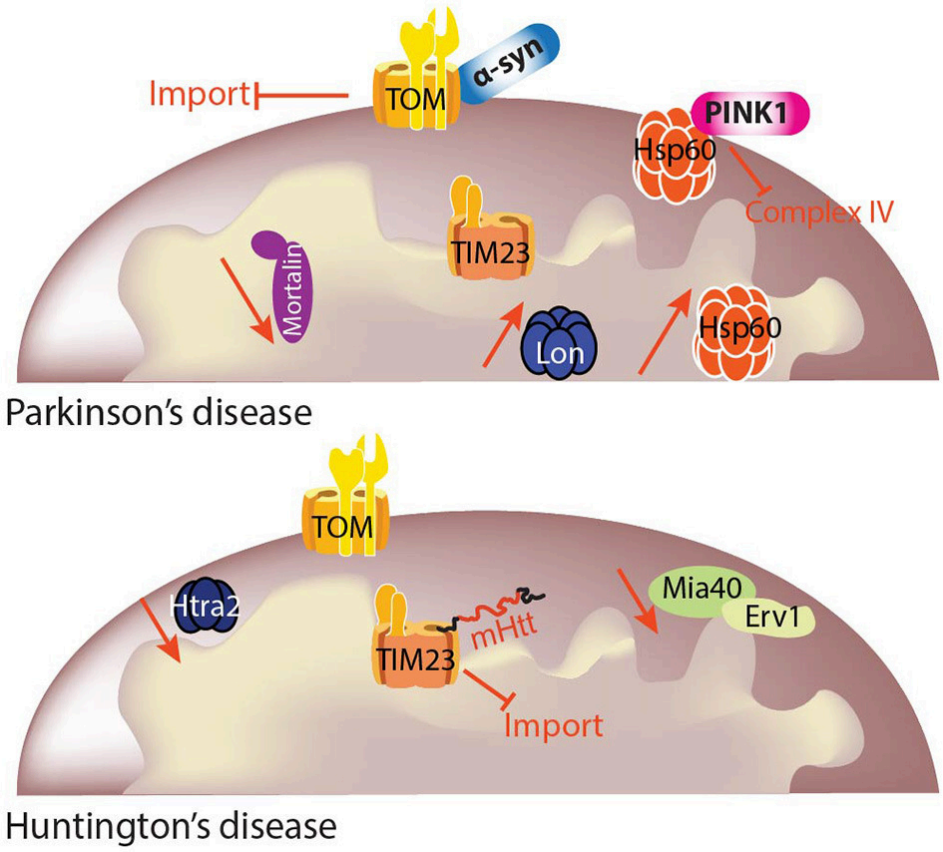
Mitochondrial protein quality control (MQC) in PD and HD. Mitochondrial function and cellular metabolism are dependent on maintenance of the mitochondrial proteome. It is critical to ensure that the mitochondrial proteome is correctly regulated depending on cellular demands, and this is achieved by protein import and protein quality control. Different import pathways and machineries exist for precursor translocation into the mitochondria. Mitochondrial precursor proteins begin their journey in the cytosol and are delivered to the translocase of the outer membrane (TOM) complex. Precursor proteins containing a positively charged N-terminal presequence are then delivered to the translocase of the inner membrane 23 (TIM23) complex for their translocation into or across the inner mitochondrial membrane in a membrane potential (Δψ)-dependent manner. Each mitochondrial compartment has its own quality control machinery. The link between MQC and neurodegenerative diseases is still under investigation, but some studies already have shown specific deficits linked to particular diseases. Although mitochondrial protein import and quality control may have been traditionally investigated independently, the overlap between the two is now clear and mitochondrial protein homeostasis cannot be sustained without both of these processes.

Several studies have linked a deficit of mitochondrial import with the accumulation of α-synuclein within the mitochondria. Most α-synuclein is soluble and resides in the cytoplasm. However, some years ago, Devi et al. (2008) reported the presence of a “cryptic” N-terminus MTS in the human α-synuclein protein sequence. They also observed an increased accumulation of α-synuclein in mitochondrial fractions from post-mortem SNpc of PD patients although which protein of the import machinery interacts with α-synuclein is still under investigation. Authors showed that α-synuclein can be translocated into the mitochondria using physiological import machinery, particularly the TOM complex (Devi et al., 2008). Another study suggested the interaction of α-synuclein with Tom40 and SAM50 (McFarland et al., 2008), while Bender et al. (2013) reported decreased Tom40 protein levels in post-mortem human midbrain samples and in the α-synuclein transgenic mouse model. Moreover, the overexpression of Tom40 in α-synuclein-accumulating cells and in the α-synuclein transgenic mouse model resulted in decreased α-synuclein levels. However, no mechanism was elucidated and import was not measured (Bender et al., 2013). On the other hand, another study recently linked α-synuclein with Tom20; the authors described an α-synuclein-specific interaction with Tom20 in nigrostriatal dopaminergic neurons of different genetic and pharmacological PD models and in human post-mortem tissue. This interaction was prevented by knocking down α-synuclein. Using *in vitro* models, oligomeric, dopamine-modified, and phosphomimetic mutant α-synuclein were able to impair protein import and induce mitochondrial dysfunction. Moreover, α-synuclein disrupts the normal Tom20-Tom22 interaction, possibly by binding to the MTS receptor site in Tom20 (Di Maio et al., 2016; Figure 3). These results tie in two pathogenic characteristics of PD: α-synuclein accumulation and mitochondrial dysfunction. However, very few reports have linked PD with mitochondrial protein import, and they seem to contradict each other, making it necessary to clarify the exact role that α-synuclein plays under normal and pathogenic conditions, and whether complex I inhibition can also impair the translocation of proteins into mitochondria.

One of the first clues linking PD to molecular chaperones was the observation of decreased mortalin levels in the SN of PD patients. Using proteomics, Jin et al. (2006) reported a significant decrease of mortalin levels in mitochondria-enriched fractions from the SN of PD patients compared to age-matched controls (Jin et al., 2006; Figure 3). Three variants in the mortalin gene (*HSP9*), two missense (R126W and P509S) and a 17 kb insertion in intron 8, were found in 3 patients from a Spanish cohort but in none of the controls, suggesting a genetic link between mortalin and PD. The missense variants consisted of an amino acid change in the actin-like ATPase domain and in the HSP peptide binding domain of the protein, both amino acids being highly conserved between HSP70 members from different species (De Mena et al., 2009). A German cohort was also screened for mortalin variants, with one variant, A476T, suggested to act as a risk factor for PD together with the previously reported variants. Cells overexpressing PD-associated variants of mortalin showed increased ROS levels after proteolytic stress compared to cells expressing wild-type mortalin. Moreover, mitochondrial dysfunction induced by knock-down of mortalin could only be rescued by wild-type mortalin but not by PD-associated variants (Burbulla et al., 2010). Some of these variants were mimicked in yeast, thus unraveling the presence of a wide range of mitochondrial alterations and further reinforcing mortalin involvement as a susceptibility factor for PD (Goswami et al., 2012). Mortalin has been found to interact with several proteins linked to genetic PD, such as DJ-1 (Jin et al., 2007; Burbulla et al., 2010), α-synuclein (Jin et al., 2007), or Parkin (Davison et al., 2009). Interestingly, PINK1 or Parkin overexpression in mortalin-knock down cells rescued the mitochondrial phenotype, thus suggesting that mortalin might also participate in the PINK1-Parkin pathway (Yang et al., 2011; Burbulla et al., 2014). However, in recent years, mortalin's role in the pathogenesis of PD has been questioned. In the screening of a cohort of 139 early-onset PD patients, only one missense change in the PD group and another in the control group were found, suggesting that mortalin variants may not be a major determinant of early-onset PD (Freimann et al., 2013). Another study of 500 PD patients and 500 controls from a Korean population revealed no significant association of *HSP9* variants with PD (Chung et al., 2017).

While the most accepted PINK1 substrates are ubiquitin and Parkin, TRAP1 was the first *in vivo* target identified for PINK1, linking mitochondrial protein quality control and PD. PD-linked PINK1 mutations (G309D, L347P, and W437X) abolished basal and oxidative stress-induced TRAP1 phosphorylation *in vitro*, thus impairing PINK1's cytoprotective effect against oxidative stress (Pridgeon et al., 2007). The authors revealed that PINK1 overexpression protects cells against oxidative stress-induced apoptosis by suppressing mitochondrial cytochrome c release; this protective action depends on the PINK1-dependent phosphorylation of TRAP1 (Pridgeon et al., 2007). Moreover, Drosophila TRAP1-null mutants present some features similar to PINK1 and Parkin null mutants; TRAP1 is able to partially rescue mitochondrial impairment in Parkin mutant flies and vice versa, suggesting that TRAP1 might work in parallel with Parkin in Drosophila (Costa et al., 2013). In human SH-SY5Y cells, TRAP1 was able to rescue mitochondrial dysfunction upon siRNA-induced silencing of PINK1 but not of Parkin (Zhang et al., 2013). Taken together, these data suggest that TRAP1 works within the PINK1-Parkin pathway to maintain mitochondrial integrity, and is also altered in the presence of PINK1 or Parkin loss-of-function mutations. TRAP1 was also decreased in A53T α-synuclein-expressing flies, resulting in an increased sensitivity to oxidative stress and motor alterations that were rescued after TRAP1 overexpression, thus providing a link between PINK1 and α-synuclein through TRAP1 (Butler et al., 2012). PINK1 also interacts with HSP60 (Rakovic et al., 2011) and its protein levels are decreased in PINK1 null cells, which was associated with complex IV deficit (Kim et al., 2012). The protease LONP1 is responsible for the degradation of oxidized proteins. MPTP-intoxicated mice displayed increase expression of LONP1 in the ventral mesencephalon concomitantly with the presence of oxidized proteins and dopaminergic cell loss. Moreover, LONP1 activity was inactivated by increased ROS levels, raising the possibility that LONP1 dysfunction represents an early event in the pathogenesis of PD (Bulteau et al., 2017). Mutations in the gene encoding HTRA2, another protease, were reported in PD patients. These mutations do not affect the localization of the protein, but instead affect its proteolytic activity, resulting in increased mitochondrial susceptibility (Strauss et al., 2005). Mice lacking HTRA2 expression develop a specific neurodegeneration of striatal neurons together with a parkinsonian phenotype (akinetic and rigid syndrome, showing a lack of coordination, decreased mobility and tremor) leading to early death, thus challenging the notion that HTRA2 is a major regulator of apoptotic cell death (Martins et al., 2004). HTRA2 phosphorylation increases its proteolytic activity in a PINK1-dependent manner, suggesting that these proteins might be part of the same stress-sensing pathway (Plun-Favreau et al., 2007). In the same way, drosophila HTRA2 mutants develop a similar phenotype to those of PINK1 and Parkin mutants, and HTRA2 was able to rescue the PINK1 phenotype, also suggesting that HTRA2 and PINK1 might act in the same pathway (Tain et al., 2009), but independently from Parkin (Whitworth et al., 2008). TRAP1 might work as a downstream effector in this pathway since HTRA2 and TRAP1 can interact with each other; however, this interaction does not involve the protease activity of HTRA2 (Fitzgerald et al., 2017). Another protease, rhomboid-7, was found to act upstream of PINK1 and HTRA2 by cleaving their precursor forms (Whitworth et al., 2008). Other studies, however, reported no association of HTRA2 variants with PD (Simón-Sánchez and Singleton, 2008; Krüger et al., 2011).

### Mitochondrial protein quality control and huntington's disease

Huntingtin fragments have been reported to be in close apposition to mitochondria in cellular and animal models of HD (Gutekunst et al., 1998; Choo et al., 2004; Orr et al., 2008) as well as in brain mitochondria of the caudate nucleus of HD patients (Yano et al., 2014) suggesting a direct role of mutant huntingtin in mitochondrial dysfunction. Localization of mutant huntingtin to brain mitochondria from mouse has been reported to impair protein import through the interaction with TIM23 complex. Importantly, mutant huntingtin association with TIM23 complex was not present with wild-type huntingtin, suggesting a role for polyglutamine domains in the interaction (Figure 3). Interestingly, import deficiency is present in highly purified synaptosomal mitochondria from presymptomatic R6/2 mice, thus indicating that the deficiency represents an early deficit in a subset of mitochondria where high energetic demands are placed, suggesting that they are more sensitive to these mutations. The rescue of mitochondrial protein import by lentiviral delivery of Tim23, Tim50, and Tim17a, prevented mitochondrial dysfunction and cell death in mutant huntingtin-expressing neurons (Yano et al., 2014). The presence of mutant huntingtin with 111 polyglutamine repeats also seems to impair the mitochondrial disulfide relay system (MDRS) in mouse striatal cells. Specifically, Napoli et al. (2013) reported a decrease in FAD-linked sulfhydryl oxidase augmenter of liver regeneration, also called Erv1, and Mia40 (mitochondrial intermembrane space import and assembly protein 40), together with one of its downstream substrates, Cox17, in total cell lysates. This deficit was concomitant with the presence of a disrupted mitochondrial morphology, lower mtDNA copy number and increased deletions, lower complex I, IV, and V activities, decreased ATP production and increased oxidative stress (Napoli et al., 2013). Importantly, MDRS is in charge of the translocation of some complex I and complex IV proteins (Mesecke et al., 2005) and small TIM chaperones (Tim8, 9, 10, and 13) (Lutz et al., 2003), some of which are part of TIM22 complex, which, in turn, is responsible for the insertion of other translocases. This suggests that an initial deficit in MDRS could lead to an altered downstream import pathway (Figure 3). However, the reported study presents some limitations such as the use of only *in vitro* models and the absence of any mechanistic description. It is true, though, that an impairment of the mitochondrial import system can hamper the translocation of proteins involved in the TCA cycle, OXPHOS, and antioxidant defenses, among others, giving rise to decreases in ATP production and ROS levels, both classical hallmarks of HD.

A missense mutation (S276C) in the protease domain of HTRA2 was discovered to be the cause of motor neuron degeneration 2, a spontaneous recessively inherited mutation that appeared in the C57BL/6J inbred background. Interestingly, this mutation leads to specific degeneration of striatal neurons (Jones et al., 2003), suggesting that those neurons are particularly vulnerable to HTRA2 deficits and indicating that this protein could play a role in HD. In a study comparing the gene expression profiles of three types of neurons expressing wild-type or mutant huntingtin, HTRA2 mRNA was specifically downregulated only in striatal neurons with mutant huntingtin (Tagawa et al., 2007). HTRA2 is downregulated in primary striatal neurons and in striatal neurons of R6/2 mutant huntingtin-transgenic mice at the presymptomatic stage, but not in the cortex or cerebellum. Moreover, the overexpression of HTRA2 in primary neurons protected against mutant huntingtin-induced cell death, while the suppression of HTRA2 renders those neurons susceptible to mutant huntingtin. These data suggest that the selective reduction of HTRA2 in striatal neurons could be linked to their selective vulnerability in HD pathology (Inagaki et al., 2008).

While it is clear that proteostasis is altered in HD, it remains uncertain whether the aggregates present are the main mediators of neuronal dysfunction. Those aggregates can have a dual role, on one hand sequestering the toxic protein, but on the other, acting as a hub to incorporate several proteins. While molecular chaperones are the first line of defense against protein aggregates, expression of expanded polyglutamine peptides leads to impaired protein folding capacity. However, no specific alteration in mitochondrial molecular chaperones has been reported so far in the context of HD.

### MtUPR: a new piece in the neurodegeneration puzzle?

As discussed above, the mitochondrial proteome is encoded by both mitochondrial and nuclear DNA. Proteostatic stress can arise for a variety of reasons: (i) when there is a high input of nuclear-encoded mitochondrial proteins that need to be folded, (ii) imbalance between the nuclear and the mitochondrial genome, or (iii) oxidative stress that modifies endogenous mitochondrial proteins. Mitochondria have developed their own mechanisms (such as the one present in the endoplasmic reticulum, ER) to respond to this proteostatic stress, called the mitochondrial Unfolded Protein Response (mtUPR). This response is a mechanism of mitochondrial to nucleus communication (Martinus et al., 1996) to activate a transcriptional program for mitochondrial homeostasis (Zhao et al., 2002).

The first characterization was made in mammalian cells and uncovered a transcriptional response characterized by the upregulation of nuclear genes encoding for mitochondrial chaperones (HSP60, HSP10, mtDnaJ) and CLPP protease (Zhao et al., 2002). However, the following studies have used *C. elegans* to further analyze the mtUPR. One of the suggested models for mtUPR activation in *C. elegans* is based on Clpp-1 (*C. elegans* homolog of CLPP protease) dependent degradation, which generates small peptides that are pumped out through the HAF-1 transporter [HAlF transporter (PGP related) family member] and contribute to the downstream signaling of mtUPR by triggering the relocalization of transcription factor DVE-1 (defective proventriculus in Drosophila homolog family member) and UBL-5 (ubiquitin-like protein 5) to the nucleus (Haynes et al., 2007, 2010; Haynes and Ron, 2010). HAF-1 is also suggested to work by modulating the stress activated transcription factor ATFS-1 import. ATFS-1 is a bZIP transcription factor normally imported into mitochondria and degraded by Lon protease. ATFS-1 has also a nuclear localization signal and upon mtUPR activation, its trafficking to mitochondria is impaired, leading to its translocation into the nucleus and subsequent transcriptional activation of several genes that are protective against mitochondrial dysfunction (Nargund et al., 2012). Therefore, ATFS-1 functions as a sensor of mitochondrial import efficiency, implying that any condition that hampers mitochondrial protein import activity could potentially activate mtUPR (Chacinska et al., 2009; Nargund et al., 2012).

While the mtUPR has been extensively investigated in *C. elegans*, it is not clear how this response occurs in mammals. In cell lines, different interventions activate CHOP (CCAAT-enhancer-binding protein homologous protein) and C/EBPβ (CCAAT/enhancer binding protein β) transcription factors, which heterodimerize, bind to target promoters on a CHOP binding site, and activate the expression of mtUPR genes (Aldridge et al., 2007). Recently, ATF5 (cyclic AMP-dependent transcription factor 5), a bZIP transcription factor, was proposed to act like ATFS-1 in *C. elegans* (Fiorese et al., 2016). Importantly, in response to mitochondrial stress in both *C. elegans* and mammals, there is an increased phosphorylation of eIF2α (eukaryotic translation initiation factor 2 α) resulting in a reduction of protein synthesis (Baker et al., 2012; Martínez-Reyes et al., 2012; Michel et al., 2015; for mtUPR review see Schulz and Haynes, 2015; Fiorese and Haynes, 2017). In summary, mtUPR is emerging as a stress response pathway, which coordinates two different compartments, the nuclear and the mitochondrial, to promote mitochondrial health.

Several pieces of evidence have linked mtUPR with PD, but they are mostly circumstantial since mtUPR regulation in mammals is not fully understood. What is clear is that parkinsonian toxins such as rotenone or MPP^+^ (complex I inhibitors), as well as paraquat (a ROS inducer), are potent mtUPR inducers, indicating that PD pathogenesis shares common alterations with mtUPR induction, thus raising the possibility that the mtUPR response is impaired in PD and worsens mitochondrial dysfunction (de Castro et al., 2010, 2011). A reduction in protein synthesis, which is one of the consequences of mtUPR activation, rescues many of the defects in flies lacking PINK1 (Liu and Lu, 2010), while Drosophila flies overexpressing a mutant OTC (ornithine transcarbamylase) protein prone to aggregation develop mitochondrial dysfunction phenotypes similar to PINK1 and Parkin mutants (Pimenta de Castro et al., 2012), further confirming the pathogenic role of protein misfolding in PD. To this end, analysis of post-mortem brains from PD patients carrying PINK1 mutations revealed enhanced levels of misfolded components of the mitochondrial respiratory chain as well as increased levels of the mtUPR marker of activation HSP60 (Pimenta de Castro et al., 2012). Another link between mtUPR and PD is mediated by the association of HTRA2 with PD. In mice, deletion of HTRA2 results in the accumulation of unfolded proteins in the mitochondria and leads to neurodegeneration (Moisoi et al., 2009). However, the role of HTRA2 mutations in familial PD remains controversial.

The mtUPR pathway is generally regarded as beneficial for cellular homeostasis, especially in response to genetic or environmental challenges; however, some reports have started to challenge this view, indicating that the mtUPR pathway could be detrimental to the cell under some circumstances. Recently, Martinez et al. (2017) studied the mtUPR pathway in a *C. elegans* model of PD, through the expression of different forms of α-synuclein. α-synuclein PD-associated variants (A53T and A30P) were able to induce mtUPR machinery, and this induction was sustained over time. Importantly, overexpression and overactivation of ATFS-1 over time, which lead to mtUPR signaling, was found to be detrimental and to induce neurodegeneration in *C. elegans* dopaminergic neurons. In addition, the co-expression of α-synuclein and overactivation of the mtUPR potentiate α-synuclein toxicity (Martinez et al., 2017). Taken together, while these observations challenge the mainstream view of mtUPR as a protective pathway, more studies are needed to fully understand the role of mtUPR in the context of PD.

So far there are no data linking HD with mtUPR. However, given the discovery that mutant huntingtin is able to block mitochondrial protein import, which is thought to be, at least in *C. elegans*, an important sensor of mitochondrial homeostasis, it is plausible that mtUPR could play a role in HD.

## Regulation of the mitochondrial network: at the crossroads of mitochondrial dynamics, mitophagy, mitochondrial biogenesis, and mitochondrial transport

**Figure 4:**
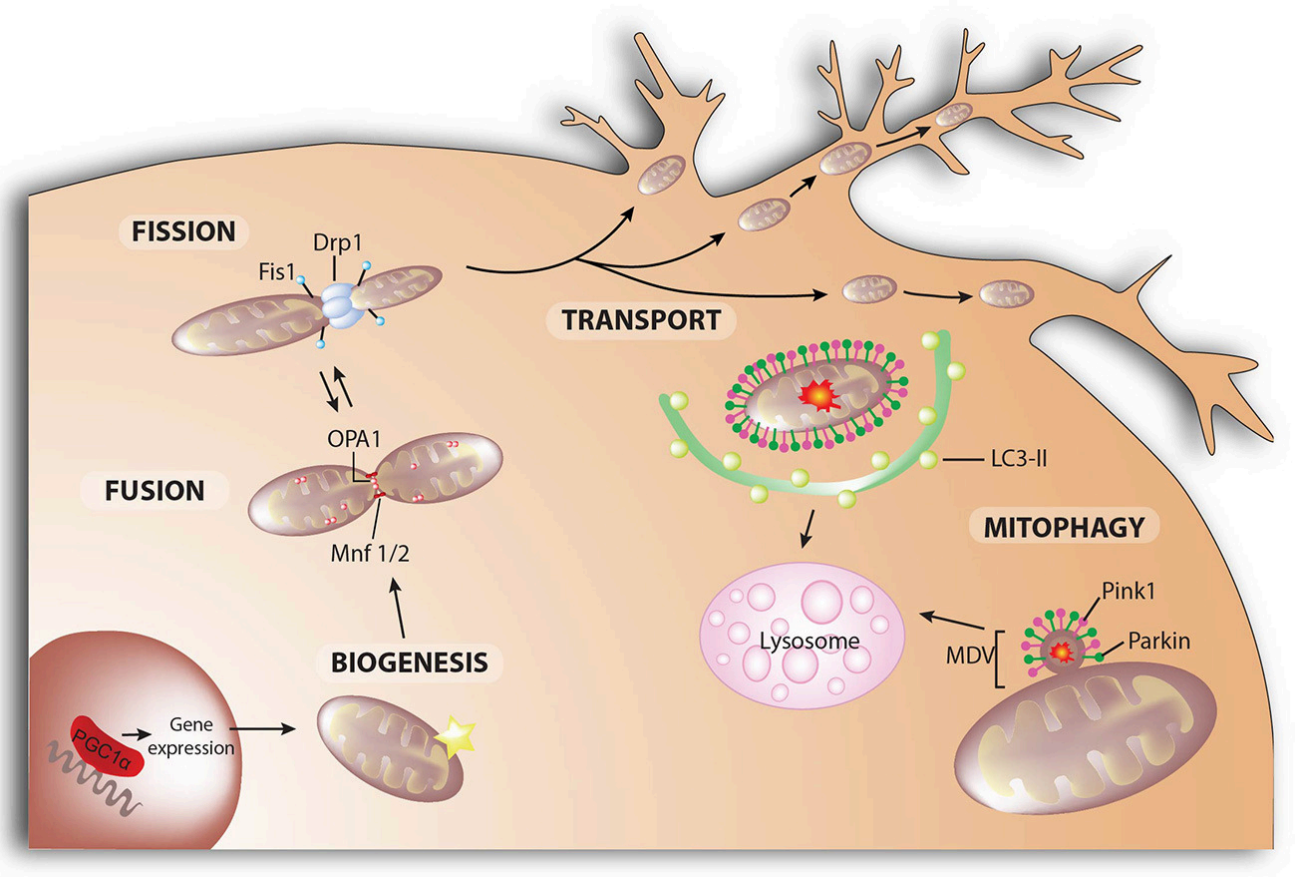
Mitochondrial dynamics. Mitochondrial fusion and fission control mitochondrial number and size. Fission is mediated by dynamin-related protein 1 (Drp1) and mitochondrial fission-1 protein (Fis1). Mitofusins (Mnf) 1 and 2 are involved in the fusion of the outer membrane, whereas protein optic atrophy type 1 (OPA1) regulates the fusion of the inner membrane. Mitochondrial biogenesis is the process by which cells increase their individual mitochondrial mass to increase the production of ATP as a response to greater energy demand. In neurons, mitochondria are recruited to subcellular compartments distant from the cell body, such axons and dendrites, by active transport along microtubules and actin filaments. Selective autophagic degradation of mitochondria (i.e., mitophagy) involves the recruitment of damaged mitochondria into a pre-autophagosome structure via a PINK1/Parkin-dependent process. When mitochondrial deterioration is mild and without global mitochondrial depolarization, local removal of mitochondrial content can be achieved by the generation of mitochondrial-derived vesicles (MDV) that bud-off from the mitochondria and contain matrix components. Both MDVs and PINK1/Parkin-targeted mitochondria are then delivered to lysosomes for degradation.

Mitochondria are dynamic organelles whose structure varies constantly from a tubular network to individual mitochondria. This mitochondrial network is controlled by the balance between various regulated processes: mitochondrial dynamics that control mitochondrial fusion and fission, *de novo* mitochondrial biogenesis, and the elimination of unwanted mitochondria by mitophagy (Ploumi et al., 2017; Figure 4). While mitochondrial fusion is believed to favor mitochondrial biogenesis by the exchange of new proteins and mtDNA between the merging organelles, mitochondrial fission is considered to be a process that isolates dysfunctional mitochondria so that they can be cleared by mitophagy.

### Mitochondrial fusion

Mitofusin 1 and mitofusin 2 (Mfn) are dynamin-like GTPases that control outer membrane fusion, whereas dynamin-like 120 kDa protein, encoded by the OPA1 gene is the dynamin-like GTPase in charge of inner membrane fusion (Mishra and Chan, 2014; Figure 4). Both Mfn have redundant roles but with certain features: (i) Mfn1, but not Mfn2, is needed for OPA1-mediated inner membrane fusion (Cipolat et al., 2004); (ii) Mfn2 mutations cause Charcot-Marie-Tooth disease type 2A, a peripheral neuropathy characterized by progressive degeneration of the peripheral nerves (Züchner et al., 2004); and (iii) in addition to its role in mitochondrial fusion, Mfn2 plays also a key role in mitochondria-ER tethering (de Brito and Scorrano, 2008). For the complete fusion of mitochondria, the inner mitochondrial membrane must fuse as well. OPA1 is a dynamin-like GTPase protein anchored to the inner mitochondrial membrane that exposes the bulk of the protein to the intermembrane space. Different OPA1 variants, determined by alternative-splicing and proteolytic cleavage, regulate the balance between fusion and fission. The processing of OPA1 by OMA1 (overlapping activity with m-AAA protease) and the i-AAA protease YME1 is central to the regulation of OPA1 activity, while the balance between long-OPA1 (L-OPA1) and short-OPA1 (S-OPA1) maintains normal mitochondrial morphology. While L-OPA1 is sufficient to mediate complete mitochondrial fusion, the activation of OMA1-dependent processing of OPA1 in the absence of YME1 leads to mitochondrial fission (Anand et al., 2014). Upon apoptotic stimuli, a complete conversion of L-OPA1 to S-OPA1 takes place, inhibiting mitochondrial fusion (Song et al., 2007). Hence, OMA1-mediated degradation of OPA1 is considered a general cellular stress response.

### Mitochondrial fission

Mitochondrial fission facilitates the segregation of damaged mitochondria from a healthy network and mitochondrial transport through neuronal processes. Dynamin-related protein 1 (Drp1) is a cytosolic protein that is recruited to the OMM, where it oligomerizes to form ring-like structures, which upon GTP hydrolysis facilitate membrane constriction (Koirala et al., 2013; Figure 4). Additional adaptors are required for Drp1 recruitment to the mitochondrial surface, such as mitochondrial fission protein 1 (Fis1), mitochondrial fission factor (Mff) and the 49 and 51 kDa mitochondrial dynamics proteins (MiD49 and MiD51) (Losón et al., 2013). Mitochondrial fission occurs at ER-mitochondrial contact sites, where ER tubules mediate constriction before Drp1 recruitment, indicating a role for ER tubules in defining division sites (Friedman et al., 2011). Less-well understood is the fission of the inner mitochondrial membrane and whether Drp1-mediated outer membrane constriction can also lead to inner membrane scission is still unknown. However, S-OPA1 fragment accumulation does favor mitochondrial fission. This discovery opens a new area of study in the field of mitochondrial fission, since further investigation is needed to understand the precise role of S-OPA1, together with its processing-peptidases, in regulating mitochondrial dynamics.

### Mitophagy

The accumulation of dysfunctional mitochondria is suggested to play a key role in the pathology of neurodegenerative diseases. Impaired mitochondria can be selectively targeted and eliminated through a process termed mitophagy. Mitophagy is a highly specialized type of autophagy that consists of three steps: recognition of the mitochondrion that needs to be cleared, formation of the autophagic membrane that surrounds the organelle, and fusion of the mito-autophagosome with the lysosome. The classical pathway to flag mitochondria for degradation involves PINK1 and Parkin and has been reviewed elsewhere (Pickrell and Youle, 2015; Martinez-Vicente, 2017; McWilliams and Muqit, 2017; Misgeld and Schwarz, 2017; Figure 4). Briefly, PINK1 is constitutively imported inside healthy mitochondria, cleaved (Whitworth et al., 2008; Jin et al., 2010; Meissner et al., 2011; Greene et al., 2012) and degraded by the proteasome (Yamano and Youle, 2013), so upon mitochondrial protein import impairment, PINK1 accumulates in the outer membrane of the dysfunctional mitochondrion and directly phosphorylates ubiquitin chains linked to the outer membrane proteins at Ser65. The presence of phospho-ubiquitins stimulates the recruitment and activation of Parkin, which is phosphorylated as well by PINK1 leading to its complete activation (Kondapalli et al., 2012; Shiba-Fukushima et al., 2012; Kane et al., 2014; Kazlauskaite et al., 2014; Koyano et al., 2014). As an E3-ubiquitin ligase, Parkin ubiquitinates many substrates with K48- and K63-linked ubiquitin chains (Ordureau et al., 2015). K63-chains recruit autophagy adaptor receptors such as sequestrome 1 (SQSTM1, also known as p62), NBR1 (neighbor of BRCA1 gene 1), OPTN (optineurin), or NDP52 (nuclear dot protein 52), while K48-chains might lead to the degradation of some outer mitochondrial proteins, which helps to control mitochondrial motility and dynamics during the process (Komander and Rape, 2012; Lazarou et al., 2015). Mitophagy receptors connect the mitochondria to be eliminated with LC3-II (microtubule-associated protein 1A/1B light chain 3B), the main component of the autophagosomal membrane (Wild et al., 2014).

Mitophagy extends well beyond the so-called PINK1-Parkin pathway. In fact, proteins in the OMM can act as mitophagy adapters under certain conditions. Nix (NIP1-like protein X, also known as BNIP3L), is induced upon hypoxia together with Bcl2/E1B 19 kDa-interacting protein 3 (BNIP3); this triggers opening of the mitochondrial permeability transition pore, depolarization, and LC3/GABARAP (GABAA receptor-associated protein) recruitment for the autophagosome formation (Zhang and Ney, 2009; Novak et al., 2010). FUNDC1 (Fun14 Domain Containing 1), an OMM-protein, has also been implicated in hypoxia-induced mitophagy. FUNDC1 phosphorylation regulates the affinity of the FUNDC1-LC3 interaction, where dephosphorylation increases this interaction and leads to mitophagy (Liu et al., 2012a). Other mitochondrial proteins have been described to act as mitophagy receptors by recruiting the autophagosomal machinery (Martinez-Vicente, 2017).

### Mitochondrial biogenesis

The need to degrade dysfunctional mitochondria must be balanced with the generation of *de novo* mitochondria to keep a healthy network (Figure 4). Mitochondrial biogenesis requires a complex coordination of the nuclear and mitochondrial expression programs. PGC-1α is the master regulator of mitochondrial biogenesis; it interacts with and coactivates several transcription factors such as nuclear respiratory factors 1 and 2 (NRF1 and NRF2), estrogen-related receptor alpha (ERRα), ying and yang 1 (YY1), myocyte enhancer factor 2C (MEF2C), peroxisome proliferator-activated receptors (PPAR), and many others coordinating mitochondrial biogenesis and oxidative metabolism (Wu et al., 1999; Scarpulla, 2006, 2011). NRF1 and NRF2 frequently work together to up-regulate the transcription of several nuclear-encoded genes with essential mitochondrial functions, and to induce the expression of mitochondrial transcription factor A (TFAM) (Virbasius et al., 1993; Scarpulla, 2008), which initiates the transcription and replication of mtDNA given its ability to bind and bend DNA without sequence specificity (Ekstrand et al., 2004). Through the regulation of TFAM levels, PGC-1α can regulate the expression of mitochondrial genes (Scarpulla, 2008).

### Mitochondrial transport

Mitochondrial transport is crucial for the distribution of mitochondria throughout the neuron, from the cell body to the presynaptic terminals, and to accomplish the resupply of newly synthesized mitochondria and mitochondrial proteins (Saxton and Hollenbeck, 2012; Schwarz, 2013; Misgeld and Schwarz, 2017; Figure 4). Large numbers of mitochondria localize at the presynaptic terminals, probably reflecting the elevated levels of ATP required for synaptic transmission and the need to regulate Ca^2+^ homeostasis during intense synaptic activity (Keating, 2007). Mitochondrial transport relies on microtubule-based motors—the anterograde kinesin-1 motor (Kif5B) and the retrograde dynein motor—attached to the mitochondria by the complex formed by Miro and a mitochondrion-kinesin linker protein, Milton (Wang and Schwarz, 2009). Stationary mitochondria are held in place by anchoring proteins, such as syntaphilin, through their interaction with microtubules (Kang et al., 2008).

### Organellar quality control and PD

Alterations in the mitochondrial fusion/fission balance have not yet been directly demonstrated in PD. However, our group and others have shown that the parkinsonian neurotoxins 6-OHDA (6-hydroxydopamine), rotenone and MPP^+^ induce mitochondrial fragmentation (fission) and cell death in neuronal cultures (Barsoum et al., 2006; Meuer et al., 2007; Gomez-Lazaro et al., 2008; Ramonet et al., 2013). Supporting a pathogenic role for mitochondrial fission, genetic inhibition of pro-fission Drp1 or pro-fusion Mfn1 or OPA1 overexpression are able to prevent cell death induced by these neurotoxins.

The potential importance of mitochondrial dynamics in PD was revealed in part by the identification of different autosomal recessive and autosomal dominant genes linked to PD. α-synuclein overexpression leads to mitochondrial fragmentation in several models, ranging from *C. elegans* to neuroblastoma cell lines (Kamp et al., 2010; Nakamura et al., 2011; Butler et al., 2012; Xie and Chung, 2012; O'Donnell et al., 2014), but no direct mechanism has been elucidated so far. It was suggested that binding of α-synuclein to sodium dodecyl sulfate micelles induces a reduction in the membrane curvature (Perlmutter et al., 2009). This observation reinforces the notion that α-synuclein-dependent mitochondrial fragmentation may be exerted through direct association with the mitochondria. Mice overexpressing A53T α-synuclein present reductions in Mfn1, Mfn2, and Drp1 protein levels in the spinal cord, which correlated with decreased mitochondrial size (Xie and Chung, 2012). However, upon Drp1 loss in mice, mitochondrial fragmentation induced by α-synuclein still occurs (Nakamura et al., 2011; Guardia-Laguarta et al., 2014). Furthermore, N-terminal disruption of α-synuclein in human induced pluripotent stem cells led to mitochondrial elongation in neurons (Pozo Devoto et al., 2017), suggesting a possible physiological role for α-synuclein in regulating mitochondrial size through its direct interaction with the fusion-fission process rather than with fusion-fission machinery.

LRRK2 protein has also been linked to mitochondrial dynamics, although its exact role remains to be clarified. Enlarged mitochondria were reported in G2019S mutant LRRK2 overexpression in Drosophila (Ng et al., 2012) and in skin biopsies from LRRK2 G2019S mutation carriers (Mortiboys et al., 2010). In Drosophila, increased mitochondrial length can be rescued by Parkin overexpression (Ng et al., 2012), suggesting that both LRRK2 and Parkin may function in the same pathway to promote fusion. LRRK2 has also been found to interact with mitofusins and OPA1 in mitochondrial membranes, while S-OPA1 levels, but not L-OPA1, are reduced in G2019S PD brains in contrast to idiopathic PD brains, where no changes in S-OPA1 are observed (Stafa et al., 2014). However, others have shown that wild-type LRRK2 interacts with Drp1 and that this interaction was exacerbated by the expression of PD-associated mutants. Moreover, LRRK2 wild-type or G2019S variant overexpression leads to mitochondrial fragmentation and clearance through the recruitment of Drp1 (Niu et al., 2012; Wang et al., 2012). Treatment with an LRRK2 inhibitor of cybrid cell lines from sporadic and LRRK2 patients (G2019S) leads to increased mitochondrial elongation and reduced phospho-Drp1 levels (Esteves et al., 2015), further supporting a role for LRRK2 in Drp1-mediated mitochondrial fission.

The PD-related proteins PINK1 and Parkin appear to control mitochondrial morphology by regulating mitochondrial fusion-fission events. However, whereas PINK1 and Parkin seem to promote mitochondrial fission and/or inhibit fusion in Drosophila, their role in mammals is more controversial (Guo, 2012; Scarffe et al., 2014). While PINK1 and Parkin functions in mitophagy have been studied extensively, their contribution to neuronal mitophagy has been challenged recently. Given that PINK1 and Parkin have been observed to be mutated in familial forms of PD, one would expect that impaired mitophagy might be a general theme in the pathogenesis of PD; however PINK1- and Parkin-knockout mice do not display neurodegeneration (Palacino et al., 2004; Gautier et al., 2008). Moreover, some groups have failed to observe Parkin recruitment in neurons following mitochondrial depolarization (Van Laar et al., 2011), or reported failure of endogenous Parkin to mediate mitophagy in neurons and cultured cells (Rakovic et al., 2013). Nonetheless, Cai et al. (2012) reported Parkin-dependent mitochondrial clearance in the somatodendritic region of primary cortical neurons upon CCCP (carbonyl cyanide m-chlorophenyl hydrazone) treatment (Cai et al., 2012). Besides, Suzuki et al. (2017) reported dysfunctional mitophagy in dopaminergic neurons derived from induced pluripotent stem cells from Parkin-mutated patients (Suzuki et al., 2017). In the MitoPark mouse model, in which there are clear mitochondrial alterations such as mitochondrial fragmentation and mitochondrial-derived aggregates, no evidence of Parkin recruitment to the defective mitochondria was observed, nor was there any effect of Parkin loss on the progression of neurodegeneration (Sterky et al., 2011). Conversely, the absence of Parkin in the Mutator mice (mice homozygous for a proofreading deficiency in DNA polymerase γ) led to a robust dopaminergic neuronal loss (Pickrell and Youle, 2015), suggesting that, at least in some cases, a basal level of mitochondrial dysfunction is needed in order to detect the detrimental effects of Parkin loss. What might be the role of PINK1-Parkin-dependent neuronal mitophagy *in vivo* is therefore still a matter of debate since; (i) most studies use proliferating cell lines, which poorly reflect the post-mitotic state of degenerating neurons; (ii) to induce mitophagy, a potent uncoupler (CCCP) is used, which inducesd a severe mitochondrial depolarization that rarely happens *in vivo*; and (iii) PINK1 and Parkin must be overexpressed in order to detect robust mitophagy. Interestingly, two *in vivo* mouse models, the mito-Keima and the mito-QC, have been recently developed (Sun et al., 2015; McWilliams et al., 2016). These models, which are based on the use of fluorescent reporter proteins, enable the visualization of mitophagy *in vivo* and might help to finally understand the *in vivo* role of PINK1-parkin-dependent mitophagy. Of note, loss of function of glucocerebrosidase and SREBF1, both proteins linked to or associated with PD, have been shown to be linked to mitophagy defects (Osellame et al., 2013; Ivatt et al., 2014).

PINK1 and Parkin have also been linked to the biogenesis of a population of MDV. Importantly, this process takes place faster than mitophagy, suggesting that such a mechanism may help to preserve the integrity of the organelle while damaged components are extracted. Parkin seems to colocalize with MDVs in a PINK1-dependent fashion upon oxidative stress, and MDVs are targeted to lysosomes for degradation independently of “typical” mitophagy mechanisms (McLelland et al., 2014). MDVs transport specific oxidized mitochondrial cargo, including subunits of the OXPHOS chain such as COXI, a transmembrane component of complex IV of the electron transport chain (McLelland et al., 2014; Sugiura et al., 2014). Interestingly, VPS35, which is linked to late-onset autosomal dominant PD, participates in the generation of some MDVs (Braschi et al., 2010).

Important advances emphasizing the contribution of impaired mitochondrial biogenesis to PD have recently been made. For downregulation of nuclear-encoded complex I genes was found associated with decreased expression of mitobiogenesis factors in PD frontal cortex, pointing at defects in mitochondrial biogenesis as another player in mitochondrial dysfunction (Thomas et al., 2012). Parkin also plays a role in the mitochondrial biogenesis pathway through the ubiquitination of Parkin Interacting Substrate (PARIS), leading to its ubiquitin-dependent degradation. PARIS is a major transcriptional repressor of PGC-1α expression, with a loss of Parkin in adult mice leading to PGC-1α downregulation due to PARIS upregulation, and dopaminergic neurodegeneration (Shin et al., 2011). The aforementioned study is particularly interesting since earlier reports failed to detect neurodegeneration in Parkin germline knock-out models. Here, there is a conditional knock-out in adult mice, which may suggest that whole body knock-out since birth may result in the development of compensatory mechanisms. The authors argue that similar compensatory mechanisms might occur in PD, accounting for the age-dependence of neurodegeneration observed in PD. PGC-1α and NRF-1 mRNAs are downregulated in the SN and striatum of PD patients, with no changes in mRNA levels of PARIS or Parkin, indicating that the upregulation of PARIS protein levels responds to a loss in the E3 ubiquitin ligase activity of Parkin (Shin et al., 2011). Decreased PGC-1α protein levels are associated with a loss in mitochondrial markers (Jiang et al., 2016). Further supporting the role of PGC-1α in PD, PGC-1α overexpression in adult conditional Parkin knock-out mice prevents dopaminergic neurodegeneration (Shin et al., 2011). Moreover, upon Parkin loss, mice present reduced mitochondrial size and number, together with structural abnormalities. These mitochondrial alterations depend on PARIS accumulation, since its loss rescues the mitochondrial mass (Stevens et al., 2015). This data further reinforces the role of the Parkin-PARIS-PGC-1α axis in mitochondrial biogenesis and homeostasis, and provide more evidence for the key role of Parkin in the regulation of mitochondrial quality control. Recently, it was shown that PINK1 cooperates with Parkin by mediating PARIS phosphorylation to control its ubiquitination and clearance by Parkin (Lee et al., 2017). PGC-1α conditional deletion in adult mice leads to dopaminergic loss in the SNpc together with a reduction of dopamine in the striatum, highlighting the role of PGC-1α in neuronal viability in adult mice (Jiang et al., 2016). PGC-1α polymorphisms are associated with increased risk and age of onset of PD (Clark et al., 2011). The tandem PINK1-Parkin also participates in mitochondrial transport regulation by mediating Miro phosphorylation and ubiquitination, which targets the protein for proteasomal degradation (Wang et al., 2011; Liu et al., 2012b). Since PINK1 stabilization in the OMM occurs in damaged mitochondria, this mechanism is thought to arrest dysfunctional mitochondria, potentially decreasing their capacity to fuse with healthy ones. PINK1 and/or Parkin loss would facilitate the trafficking of dysfunctional mitochondria toward the axon terminals, where they could hamper energy production. Recent work has linked the α-synuclein A53T mutation with an altered balance in anterograde and retrograde transport (Pozo Devoto et al., 2017), while the LRRK2 R1441C variant is associated with impaired neuritic mitochondrial transport in SH-SY5Y cells (Thomas et al., 2017).

### Organellar quality control and huntington's disease

In HD, the balance between fusion and fission is aberrantly shifted toward fission, which is associated with increased levels of Drp1 and Fis1 mRNA and decreased mitofusins in striatal and cortical regions (Kim et al., 2010; Shirendeb et al., 2011). These changes in key proteins controlling the mitochondrial fission/fusion balance are translated into mitochondrial fragmentation in HD human tissue and mouse models (Liot et al., 2009; Shirendeb et al., 2011). The mechanism leading to exacerbated mitochondrial fission in the presence of mutant huntingtin seems to involve a direct interaction with Drp1. In human post-mortem samples and in transgenic mice lines, mutant huntingtin interacts with Drp1 with a higher affinity than that of the wild-type huntingtin. This interaction leads to increased Drp1 enzymatic activity and mitochondrial fragmentation. The transfection of a dominant-negative Drp1 mutant (Drp1 K38A) leads to elongated and uniformly distributed mitochondria and protects against ATP loss and cell death, further supporting a role for Drp1 in mitochondrial fragmentation (Wang et al., 2009; Song et al., 2011; Shirendeb et al., 2012). Consistent with this hypothesis, Drp1 GTPase activity was increased in the cortex of HD patients (Shirendeb et al., 2012). Furthermore, blocking the interaction of Drp1 with Fis1, which inhibits mitochondrial fission, rescues striatal neuronal loss, improves motor activity and reduces mortality in R6/2 HD mice (Guo et al., 2013), suggesting that shifting the balance toward mitochondrial fusion could have a positive impact on disease progression. Brains of HD patients exhibit increased levels of S-nitrosylated Drp1 (Haun et al., 2013), a post-translational modification reported to enhance its GTPase activity (Cho et al., 2009). Since mutant huntingtin increases nitric oxide production, it is hypothesized that mutant huntingtin's interaction with Drp1 enhances its nitrosylation, thereby increasing mitochondrial fragmentation in HD (Haun et al., 2013).

Huntingtin protein plays an important role in the cargo recognition step in selective autophagy (Ochaba et al., 2014); the presence of the polyglutamine tract can affect the efficiency of this step and thus lead to impaired clearance of damaged mitochondria by mitophagy. Initial work by Martínez-Vicente et al. described the presence of “empty autophagosomes” in HD cells, suggesting that mutant huntingtin somehow impairs the cargo sequestration step, leading to inefficient cargo loading with lipid droplets and mitochondria being excluded from autophagic vacuoles (Martinez-Vicente et al., 2010). Huntingtin acts as a scaffold to bring together different proteins needed for autophagy to take place, such as p62 and ULK1 (unc-51 like autophagy activating kinase 1), which is normally inhibited by its association with mTORC1 (mammalian target of ramapycin complex 1). Upon exposure to different stresses, UKL1 is released from mTOR inhibition and interacts with huntingtin in a kinase-active form (Rui et al., 2015). It is likely, therefore, that the polyglutamine tract affects huntingtin's ability to associate with cargo receptors and autophagy machinery. While some studies have suggested that the presence of undigested mitochondria could stem from disturbances in autophagosomal transport, which is required for lysosomal fusion to autophagosomes (Wong and Holzbaur, 2014), others reported decreased LC3-mitochondria colocalization, arguing in favor of decreased mitophagy due to an impairment in the targeting of defective mitochondria to autophagosomes (Khalil et al., 2015). Interestingly, PINK1 overexpression could partially rescue the mitophagy defect in mouse striatal cells expressing mutant huntingtin (Khalil et al., 2015). Recent work has also linked mutant huntingtin with the impairement of a new form of micro-mitophagy mediated by GAPDH (glyceraldehydes-3-phosphate dehydrogenase) association with damaged mitochondria upon oxidative stress (Hwang et al., 2015). All in all, the ability of mutant huntingtin to interfere with different quality control process at the level of the whole mitochondria organelle prevents the correct elimination of damaged mitochondria, which potentially amplifies the deleterious cascade.

Studies on axonal transport in several *in vitro* models of HD reported impaired mitochondrial trafficking (Trushina et al., 2004; Chang et al., 2006; Orr et al., 2008). Wild-type huntingtin is involved in fast axonal trafficking in mammals, and this function has been attributed to its association with huntingtin-associated protein-1 (HAP1) (Gutekunst et al., 1998). HAP1 is an essential neuronal protein that interacts with dynactin p150 (Engelender et al., 1997) and kinesin light chain (McGuire et al., 2006). In contrast, mutant huntingtin leads to trafficking abnormalities (Gunawardena et al., 2003), but the exact mechanism for this remains unknown. One possibility is that mutant huntingtin leads to abnormal interactions with HAP1, leading to impaired vesicular and organellar trafficking. Moreover, data from mouse neurons and human HD-affected brain suggests that large mutant huntingtin aggregates impair neuronal trafficking by sequestering wild-type huntingtin and motor proteins from soluble pools (Trushina et al., 2004). This trafficking defect also affects mitochondrial motility and appears to correlate with glutamine length (Trushina et al., 2004), leading mitochondria to accumulate adjacent to aggregates and become immobilized (Chang et al., 2006). Moreover, some polyglutamine-containing N-terminal huntingtin fragments, caused by proteolytic cleavage, can associate with mitochondria and, *in vitro*, interfere in its association with microtubule-based transport proteins, thus illustrating another mechanism by which mutant huntingtin can impair mitochondrial trafficking (Orr et al., 2008). More recently, mitochondrial trafficking was studied in primary hippocampal neurons from bacterial artificial chromosome mouse expressing full length human mutant huntingtin (BACHD mice) (Shirendeb et al., 2012). The authors found a decreased number of mitochondria moving anterogradely, together with increased numbers of mitochondria in the cell soma. Neuronal processes were, moreover, devoid of mitochondria, thus further documenting the aberrant mitochondrial transport associated with mutant huntingtin expression.

As discussed above, early-stage HD patients present decreased PGC-1α mRNA levels in the striatum but not in the hippocampus or cerebellum. Expression data from HD caudate tissue showed reduced expression of 24 out of 26 PGC-1α target genes (Cui et al., 2006; Weydt et al., 2006), while a splice variant of PGC-1α, which leads to a 38 kDa protein that complements, overlaps or prolongs PGC-1α full length action, has been found to be severely altered in human HD brain and myoblasts, as well as in mouse and cellular HD models (Johri et al., 2011; Török et al., 2015). Recently, a coding variant in PGC-1α was found to be associated with the age of onset of motor symptoms in men carrying the HD mutation (Weydt et al., 2014). Moreover, a correlation exists between PGC-1α reductions and mitochondrial reductions in HD post-mortem brain (Kim et al., 2010). In line with this, PGC-1α null mice develop a neurological phenotype consistent with neurodegeneration, together with spongiform lesions predominantly in the striatum (Lin et al., 2004). Pharmacologic activation of PGC-1α expression resulted in improved behavior, improved survival and reduced brain, muscle, and brown adipose tissue pathology in R6/2 transgenic and full-length huntingtin mouse models of HD (Johri et al., 2012; Chandra et al., 2016). These observations provide further support of the key role that alterations in the PGC-1α pathway play in mitochondrial dysfunction in HD. cAMP signaling is a key activator in PGC-1α transcription, promoting the binding of cAMP response element (CRE)-binding protein (CREB) or activating transcription factor-2 to a conserved DNA response element in the PGC-1α promoter. Mutant huntingtin interferes with the CREB/TAF4 transcriptional pathway in striatal neurons, thus repressing the CRE-mediated transcription of PGC-1α (Cui et al., 2006; Weydt et al., 2006). Levels of TORC (Transducers of Regulated CREB Activity), a CREB coactivator, were decreased in the striatum of HD patients, mice and cellular HD models (Chaturvedi et al., 2012).

## Conclusion

**Figure 5:**
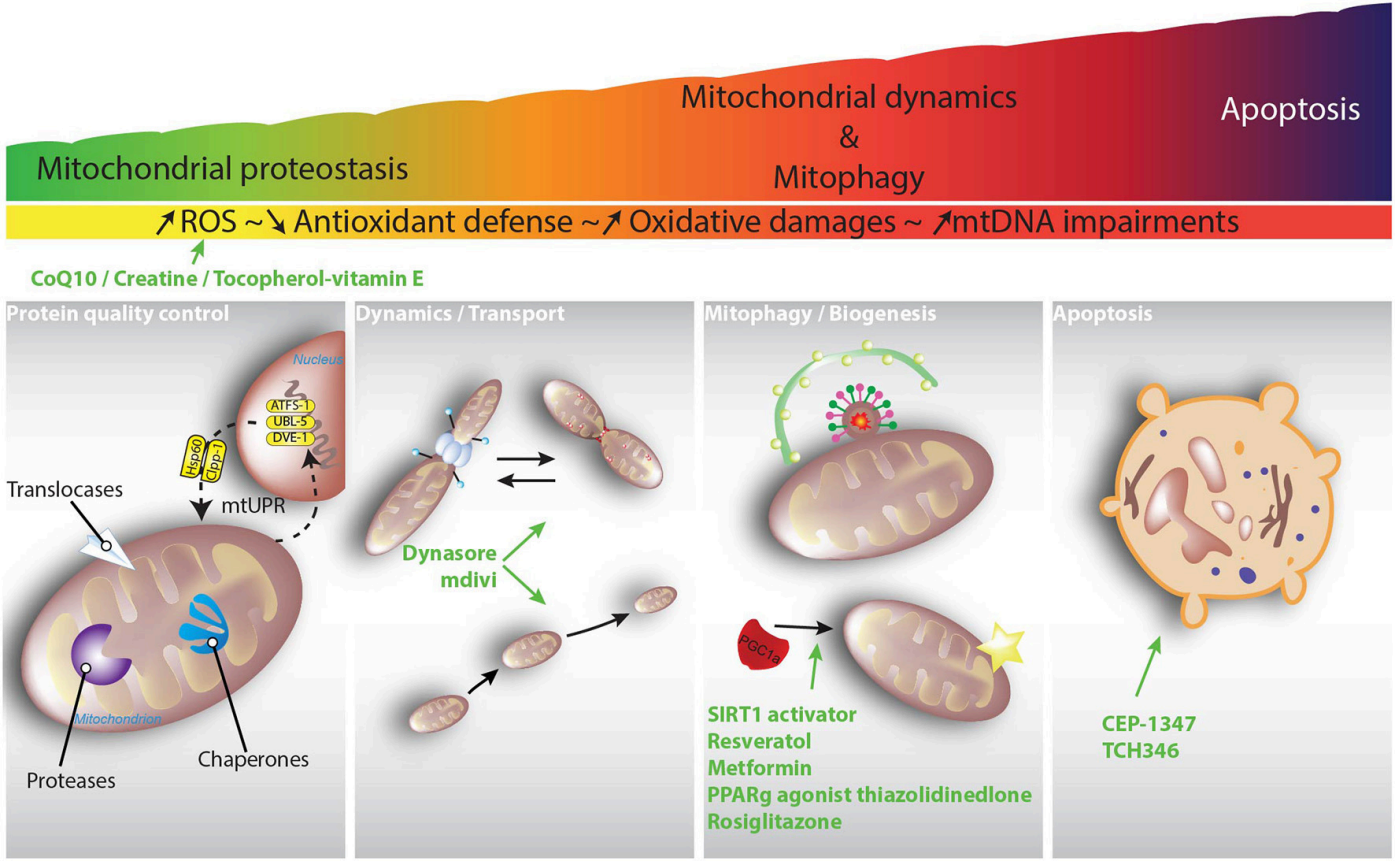
Schematic diagram depicting an hypothesized scenario responsible for mitochondrial dysfunction in PD and HD. ROS are continuously produced *in vivo* by all body tissues. The presence of mitochondrial translocases, chaperones and proteases within the mitochondrial matrix and intermembrane space acts as a first line of defense against unfolded and oxidized soluble proteins. Mitochondria unfolded response (mtUPR) is believed to be activated by cytosolic transcription factors (ATFS-1, UBL-5, DVE-1). Once inside the nucleus, these transcription factors promote the upregulation of genes involved in mitochondrial proteostasis. To maintain a healthy network, mitochondrial are dynamic and can fuse, divide, and move. Once the accumulation of mitochondrial defects exceeds a threshold, patches of mitochondria can be removed through the generation of mitochondrial-derived vesicles (MDVs), which transit to the lysosome. Upon complete mitochondrial dysfunction, the entire organelle can be targeted to the autophagosome via so-called mitophagy. When none of the rescue strategies are able to restore mitochondrial function, the cell enters its ultimate destiny, apoptosis. Clinical trials using general caspase inhibitors such as CEP-1347 and TCH346 failed, maybe because it was too late in the process to intervene. Other compounds were tested in clinical trials, from fission inhibition using mdivi to PPARγ activation, but none were able to show clear treatment benefits. Interestingly, the role of mitochondrial quality control (MQC) in neuronal health is a recently growing subject for investigation, indicating that MQC might be central to maintain healthy mitochondria. However, until now, no novel therapeutic strategy specifically targeting MQC has been developed. It is clear that future research focusing on understanding MQC is needed to develop better pharmacological interventions.

Mitochondria are essential organelles for the maintenance of neuronal homeostasis. The importance of functional mitochondria to neurons is highlighted by the fact that situations leading to mitochondrial dysfunction are often associated with neurodegenerative diseases. Many of these diseases manifest later in life, where mitochondria seem to be less functional (Grimm and Eckert, 2017). Mitochondria are at the center of the free radical theory of aging by being both a source and target of ROS. As discussed in this review, the imbalance between ROS production and antioxidant defense in neurodegenerative diseases leads to protein, lipid and DNA oxidation, which in turn affect mitochondrial function. It was previously considered that when ROS levels exceeded a pathological threshold, this may trigger cell death by apoptosis (Figure 5). However, recent developments highlight the increasing potential of mitochondria to defend themselves against various threats (Perier et al., 2010, 2013). Different levels of quality control co-exist within mitochondria to detect and repair defects that affect mitochondrial performance, before the point of inescapable cell death is reached: (i) novel, critical roles of mitochondrial proteases have emerged in neural physiology and pathology and (ii) mitochondrial dynamics play an important role in maintaining a healthy organelle population. Thus, impairments in these quality control systems may lead to the accumulation of defective mitochondria, as well as inefficient mitochondrial transport and distribution, leading to synaptic and neuronal degeneration. However, the more our understanding of protein quality control activities in mitochondria continues to expand, the more questions that are raised. How are all the mitochondrial quality control mechanisms, at the protein and the organelle levels, inter-connected? As mechanisms that flag defective mitochondria are thought to be similar, are these pathways temporarily interlinked and is there crosstalk between them to coordinate mitochondrial homeostasis?

Despite the many therapeutic advances in PD and HD, no agent has yet been established that has neuroprotective effects in either disease. Although apoptosis is potentially a terminal pathway relevant to both PD and HD, therapeutic targeting of executioner caspases, despite initially high expectations due to the availability of pharmacological caspase inhibitors, has failed to prevent neurodegeneration in clinical trials (Parkinson Study Group PRECEPT Investigators, 2007; Huntington Study Group DOMINO Investigators, 2010; Figure 5). Acting before the terminal phase is reached might represent an effective neuroprotective therapy that slows or stops disease progression and prevents the development of cumulative disability (Bové et al., 2014). Some compounds, which target mitochondrial dysfunction and mitochondrial quality control, have shown beneficial effects in mouse models of neurodegenerative diseases and show great promise for treating patients (Figure 5). Our current knowledge regarding mitochondrial quality control continues to evolve, opening novel and exciting research paths that will likely help to develop clinically effective therapeutic applications to prevent or combat neurodegenerative diseases.

## Author contributions

SF-I and CP wrote the manuscript. All authors contributed to manuscript revision, read and approved the submitted version.

### Conflict of interest statement

The authors declare that the research was conducted in the absence of any commercial or financial relationships that could be construed as a potential conflict of interest.
